# Clinical Use of Cerebral Microdialysis in Patients with Aneurysmal Subarachnoid Hemorrhage—State of the Art

**DOI:** 10.3389/fneur.2017.00565

**Published:** 2017-11-03

**Authors:** Raimund Helbok, Mario Kofler, Alois Josef Schiefecker, Maxime Gaasch, Verena Rass, Bettina Pfausler, Ronny Beer, Erich Schmutzhard

**Affiliations:** ^1^Neurological Intensive Care Unit, Department of Neurology, Medical University of Innsbruck, Innsbruck, Austria

**Keywords:** subarachnoid hemorrhage, neuromonitoring, cerebral microdialysis, brain metabolism, treatment

## Abstract

**Objective:**

To review the published literature on the clinical application of cerebral microdialysis (CMD) in aneurysmal subarachnoid hemorrhage (SAH) patients and to summarize the evidence relating cerebral metabolism to pathophysiology, secondary brain injury, and outcome.

**Methods:**

*Study selection*: Two reviewers identified all manuscripts reporting on the clinical use of CMD in aneurysmal SAH patients from MEDLINE. All identified studies were grouped according to their focus on brain metabolic changes during the early and subacute phase after SAH, their association with mechanisms of secondary brain injury and outcome.

**Results:**

The review demonstrated: (1) limited literature is available in the very early phase before the aneurysm is secured. (2) Brain metabolic changes related to early and delayed secondary injury mechanisms may be used in addition to other neuromonitoring parameters in the critical care management of SAH patients. (3) CMD markers of ischemia may detect delayed cerebral ischemia early (up to 16 h before onset), underlining the importance of trend analysis. (4) Various CMD-derived parameters may be associated with patient outcome at 3–12 months, including CMD-lactate-to-pyruvate-ratio, CMD-glucose, and CMD-glutamate.

**Conclusion:**

The clinical use of CMD is an emerging area in the literature of aneurysmal SAH patients. Larger prospective multi-center studies on interventions based on CMD findings are needed.

## Introduction

First reports on monitoring of brain metabolism using cerebral microdialysis (CMD) in patients with subarachnoid hemorrhage (SAH) date back to the year 1992 (1). Despite the long-standing availability of this technique, recommendations for treatment decisions based on CMD monitoring have just been recently published as a consensus statement by clinical experts (2).

Cerebral microdialysis has improved our understanding of pathophysiological mechanisms of early and delayed brain injury in SAH patients by providing metabolic information derived from brain tissue on a cellular level, in addition to intracranial pressure (ICP), cerebral perfusion pressure (CPP), cerebral blood flow (CBF), brain tissue oxygen tension (P_bt_O_2_), and electrographic monitoring (electroencephalography and electrocorticography). So far, changes in brain metabolism have been associated with known complications after SAH and may also help to detect impending secondary brain injury early or before they have evolved into irreversibility. Moreover, abnormalities in CMD-derived parameters have been linked to poor brain tissue and functional outcome and may therefore be integrated in the multimodal approach of neuroprognostication. In addition, the effect of commonly applied pharmacological and non-pharmacological treatments on brain metabolism can be studied on an individual level, thereby enhancing the concept of personalized medicine in neurocritical care patients.

### Microdialysis Methodology

The principle of CMD is to mimic a capillary blood vessel in the brain for the assessment of local cerebral metabolism (3). A tubular semi-permeable membrane on the tip of the CMD catheter is perfused with an isotonic or colloid-enriched fluid that equilibrates with the extracellular space by simple diffusion. All molecules small enough to pass the membrane follow their electro-chemical gradient into the tube. Established catheters have a membrane length of 1 cm and pore sizes of either 20 or 100 kDa, which do not show differences in the recovery of small molecules (4). A perfusion speed of 0.3 µl/min is recommended in clinical use, which leads to a relative recovery rate (dialyzate concentration divided by true concentration) of about 70% for the most commonly assessed molecules (5).

**Figure 1:**
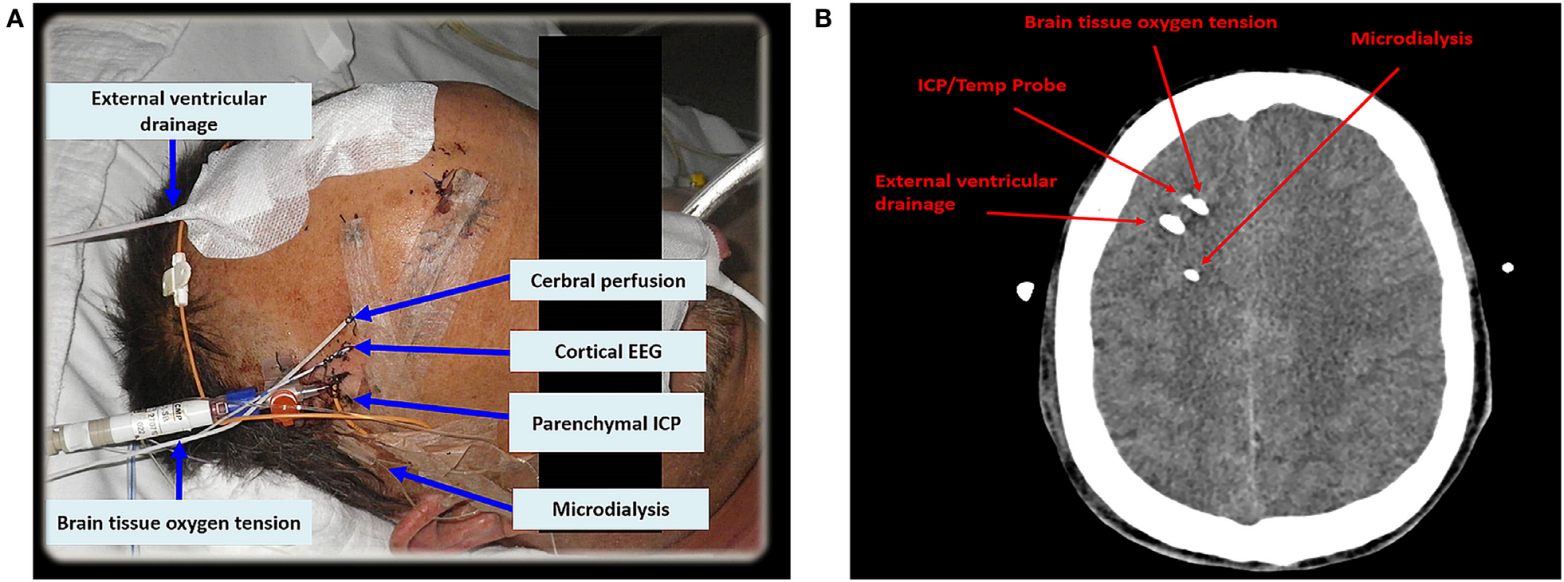
In **(A)**, multimodal neuromonitoring catheters are tunneled in a patient with subarachnoid hemorrhage. **(B)** Shows neuromonitoring catheters placed in the white matter on an axial computed tomography. EEG, electroencephalography; ICP, intracranial pressure; Temp, temperature.

Criteria for CMD monitoring are not well defined. It can be used as part of the “multimodal neuromonitoring bundle” (Figure 1A) in ventilated (“poor-grade”) patients or in patients with a secondary neurological deterioration. As a primary monitoring device, the probe should be placed in the frontal lobe (anterior/middle cerebral artery watershed), ipsilateral to the ruptured aneurysm, or the maximal blood clot load. When used in patients with secondary deterioration, location can also be guided by local practice to identify tissue at risk (for example, by CT perfusion or transcranial ultrasound) (2, 6). The catheter can be inserted into the brain tissue either by using a cranial access device (bolt) or by tunneling (Figure 1A), penetrating the skull *via* a craniotomy (Figure 1B) or a twist drill hole. The dialyzate of the first hours should be discarded due to the insertion trauma and dilution effect of the flush sequence filling the system.

### Interpretation of Data

**Figure 2:**
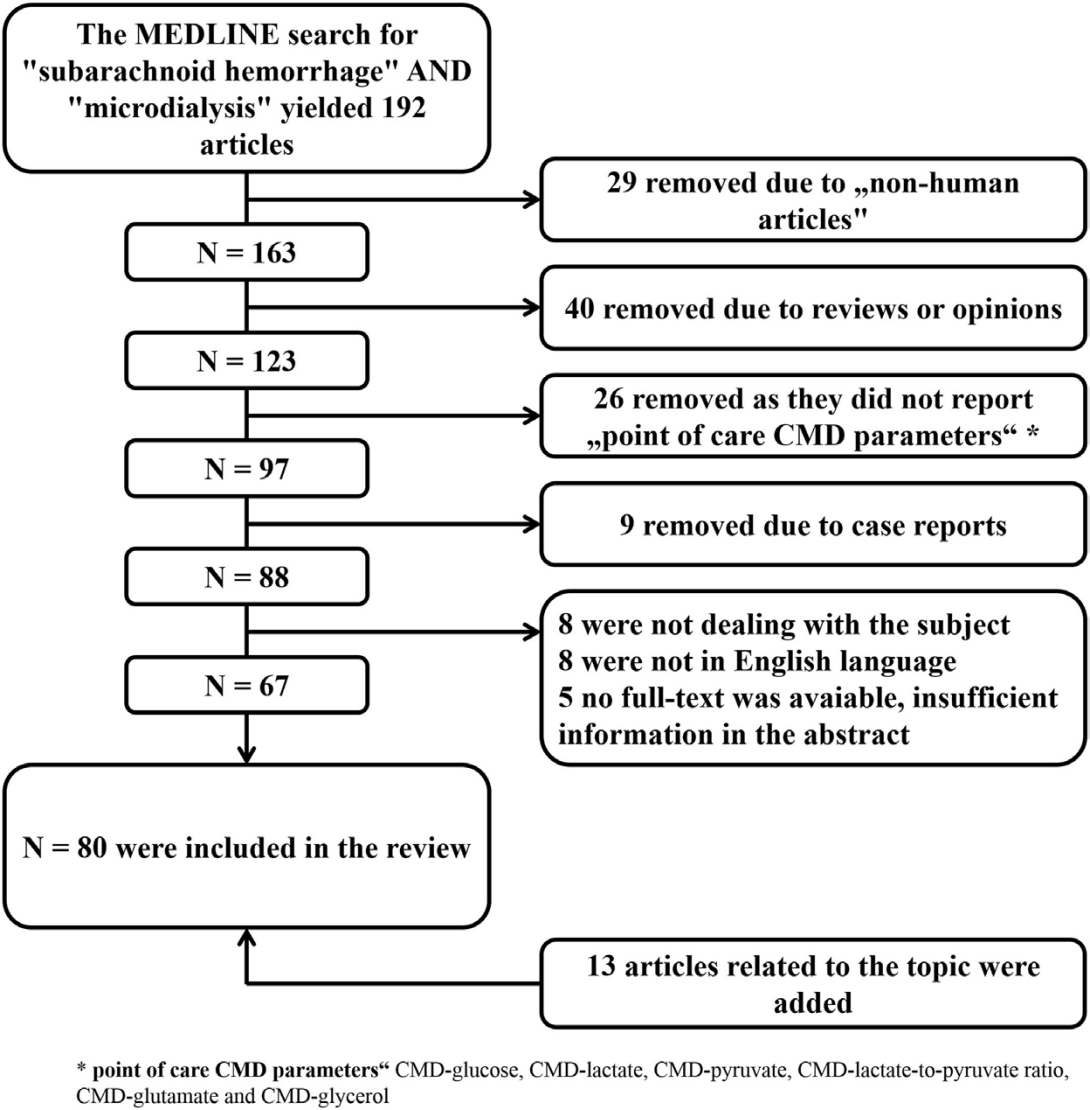
Literature search with selection of articles included in the review. CMD, cerebral microdialysis.

Concentrations of CMD parameters represent the local metabolic environment surrounding the membrane and cannot be extrapolated to other regions of the brain. Knowledge of catheter location is therefore mandatory for data interpretation. The gold tip of the CMD probe is visible on head CT (Figure 2B), thus its exact location in the brain can be determined and classified by the monitored tissue (gray vs. white matter), lobe, or by the spatial relation to focal pathologies (intralesional/perilesional vs. radiologically normal-appearing brain tissue). The dependency of molecular concentrations on probe location argues for the interpretation of temporal dynamics (trend analysis) in addition to absolute values. Calculating ratios of different CMD parameters (e.g., CMD-lactate-to-CMD-pyruvate-ratio, CMD-LPR) creates variables independent of absolute concentrations and recovery.

The dialyzate is sampled into microvials and analyzed for point of care parameters including CMD-glucose, CMD-lactate, CMD-pyruvate, CMD-glutamate, and CMD-glycerol at the patient’s bedside. A measurement interval of 1 h is commonly used in clinical practice.

Glucose is an important energy substrate for neuronal tissue. Its concentration in the brain depends on systemic supply, diffusivity in the brain tissue, and local consumption. In the process of aerobic glycolysis, it is metabolized to pyruvate and further converted into acetyl-coenzyme A, which is used for mitochondrial energy production. Under conditions of brain tissue hypoxia or mitochondrial dysfunction, pyruvate is fermented into lactate. The LPR reflects the cytoplasmatic redox state and is a marker of anaerobic metabolism and/or mitochondrial dysfunction. The concept of mitochondrial dysfunction arose from observations of impaired cerebral energy metabolism despite normal perfusion and substrate availability. The underlying pathophysiological mechanisms are not sufficiently elucidated. CMD-glutamate has fewer clinical implications, however, elevated concentrations of this excitatory neurotransmitter are considered to be a marker of ischemia and excitotoxicity. Glycerol is a component of neuronal cell membranes, thus CMD-glycerol concentrations are a surrogate marker of cell membrane damage, e.g., under conditions of hypoxia or ischemia.

## Materials and Methods

The aim of this review is to summarize the current knowledge of this technique in the critical care management of SAH patients and to discuss its limitations. A MEDLINE search was performed to identify all studies reporting on the clinical use of CMD in aneurysmal SAH patients. The selection process is summarized in Figure 2. All identified studies were grouped according to their focus on the brain metabolic changes during the early and subacute phase after SAH, their association with mechanisms of secondary brain injury and outcome.

### Definitions

The early phase was defined as the first 72 h after SAH, commonly referred to as “early brain injury” (EBI) (7). Pathological threshold values of parameters commonly given in the SAH literature are CMD-glucose < 0.7 mmol/l (referred to as neuroglucopenia), CMD-lactate > 4 mmol/l, CMD-pyruvate < 120 μmol/l, CMD-glutamate > 10 μmol/l, CMD-glycerol > 50 μmol/l, and CMD-LPR > 40. A CMD-LPR > 40 is referred to as metabolic distress. Recently, the pattern of mitochondrial dysfunction was defined as CMD-LPR > 30 together with CMD-pyruvate levels > 70 μmol/l. Important metabolic profiles in SAH patients are shown in Table 1.

**Table 1:** Summary of brain metabolic patterns using CMD in SAH patients.

	CMD-glucose	CMD-lactate	CMD-pyruvate	CMD-LPR	CMD-glutamate	CMD-glycerol
Acute focal neurological deficits	↓ to ↓↓	↑↑	n/a	↑↑	↑↑	↑↑, mainly on days 1–2 after subarachnoid hemorrhage

Global cerebral edema	↓ or no difference	↑	↓↓ or ↑ (metabolic distress or hypermetabolism)	↑↑ or ↑	n/a	n/a

Delayed cerebral ischemia	↓↓, decreasing 12–16 h before DCI	↑↑, early, sensitive, but not specific	↓ to ↓↓, rarely independently reported	↑↑, increasing 12–16 h before DCI	↑ to ↑↑, early and sensitive	↑ to ↑↑

Mitochondrial dysfunction	Within normal range	↑↑	Within normal range	↑ to ↑↑	↑ to ↑↑	↑ to ↑↑

Poor outcome	↓↓	↑ to ↑↑, unspecific	↓↓, no increase to normal values	↑↑	↑↑	↑

*A single arrow (↑/↓) indicates increased or decreased values compared to normal levels or the control group. Double arrows (↑↑/↓↓) indicate that values are above/below pathologic thresholds (CMD-glucose < 0.7 mmol/l, CMD-lactate > 4 mmol/l, CMD-pyruvate < 120 μmol/l, CMD-LPR > 40, CMD-glutamate > 10 μmol/l, CMD-glycerol > 50 μmol/l)*.

*CMD, cerebral microdialysis; n/a, no data available*.

## Results

### The Clinical Use of CMD in the Acute Phase after SAH

The initial phase after SAH is commonly referred to as “early brain injury” and comprises the first 72 h after the bleeding (7), which is pathophysiologically related to, but temporally separated from the subsequent occurrence of delayed cerebral ischemia (DCI). EBI is the result of the initial hemorrhage leading to a cascade of ischemic injury, global brain swelling and early mitochondrial dysfunction, as well as pressure-related side effects due to parenchymal hematoma or early hydrocephalus.

### Procedural Monitoring

One of the most feared complications early after SAH is aneurysm rebleeding, with the highest risk of occurrence within the first 24 h (8). Importantly, in most studies, metabolic monitoring using CMD started after the aneurysm had been secured (time to monitoring is given in Tables 3–8). While no CMD data are available during coiling, several studies have investigated changes in cerebral metabolism during aneurysm surgery (Table 2) (9–13). Commonly used CMD sampling intervals between 10 and 60 min seem to be too imprecise to depict periprocedural changes in brain metabolism (9–11), although increases in CMD-LPR and CMD-glutamate levels following prolonged artery clipping and ischemic complications were reported (9, 10). The largest study, including 38 aneurysmal SAH patients, found no significant differences in metabolic markers of brain tissue ischemia during transient artery occlusion (median occlusion time was 14 min) (11). In two patients with a prolonged occlusion time of more than 30 min, a pronounced increase of CMD-glutamate was observed (11). The feasibility of rapid-sampling microdialysis (obtaining values up to every 30 s) was investigated during temporal lobe retraction and transient artery occlusion (13). While metabolic changes reached a maximum after 3–10 min during temporal lobe retraction, increasing CMD-lactate and decreasing CMD-glucose levels were observed until clip removal during artery occlusion (13). A higher sampling frequency in CMD monitoring may help to define a threshold for ischemia to prevent irreversible tissue damage during aneurysm obliteration.

**Table 2:** Brain metabolism during aneurysm surgery.

Reference	Study type	Number of patients with SAH	Patient characteristics	Monitoring period	Probe location	Study aim	Main microdialysis findings
(13)	Single-center, prospective, observational	8	WFNS grade I *n* = 5, II *n* = 3	Intraoperative. A CMD catheter was inserted immediately after opening the dura	Territory of the parent artery of the aneurysm	Detecting adverse metabolic events during aneurysm surgery using rapid-sampling microdialysis	During temporal lobe retraction, CMD-lactate levels increased (+0.66 mmol/l) and CMD-glucose levels decreased (−0.12 mmol/l). The peak of these changes was observed after 3–10 min, despite continued retraction. During temporary artery clipping, CMD-lactate levels increased (+0.73 mmol/l) and CMD-glucose levels decreased (−0.14 mmol/l). These changes reached their maximum right before clip removal
(10)	Single-center, prospective, observational	5/12	“Preselected on the basis of anticipated difficulty in surgery”	Intraoperative	Cortical, territory of the parent artery of the aneurysm	Studying amino acid concentrations during periods of cerebral ischemia	CMD-glutamate levels increased between 2.7- and 8.1-fold during ischemic intraoperative complications. No statistical analysis was performed
(9)	Single-center, prospective, observational	10/15	n/a	Intraoperative	Cortical, territory of the parent artery of the aneurysm	To assess metabolic changes during temporary artery clipping	The CMD-LPR ranged from 32 to 65. Clipping <3 min was not followed by an increase in CMD-LPR (42–43). Prolonged clipping was followed by a pronounced increase in CMD-LPR in 2 cases (24–50 and 60–70). No statistical analysis was performed
(11)	Single-center, prospective, observational	38/46	WFNS grade. Poor (III, IV, V) in 18 patients, 7 aneurysms were larger than 25 mm	Intraoperative	Frontal or parietal lobe ipsilateral to the aneurysm	To investigate potential episodes of cerebral ischemia during aneurysm surgery	Temporary artery clipping (median duration 14 min) was not associated with significant changes in brain metabolism. In 2 patients, who post-operatively developed cerebral infarction, clipping for longer than 30 min was associated with a significant CMD-glutamate increase (2–25 µmol/l in 1 patient)
(12) (abstract only)	Single-center, prospective, observational	10/16	“Complex aneurysm surgery”	Intraoperative	n/a	To investigate cerebral metabolic changes during temporary internal carotid artery clipping	Minimal decreases in brain tissue oxygen tension were not associated with metabolic changes, while more pronounced decreases were associated with an increase in CMD-LPR. Prolonged occlusions (42 min) were associated with an increase in CMD-glutamate levels. No statistical analysis was performed

*WFNS, world federation of neurological societies; CMD, cerebral microdialysis; n/a, data not available; LPR, lactate-to-pyruvate-ratio*.

**Table 3:** The clinical use of CMD in the acute phase after SAH.

Reference	Study type	Number of patients with SAH	Patient characteristics	Monitoring period	Probe location	Study aim	Main microdialysis findings
(14)	Single-center, prospective, observational	26	Hunt and Hess grade II *n* = 2 (7.7%), III *n* = 6 (23.1%), IV *n* = 2 (7.7%), V *n* = 16 (61.5%)	Monitoring was started 22 h (median) after SAH. Data of the following 144 h are reported	Frontal, ipsilateral to the aneurysm; classified as normal-appearing or perilesional brain tissue	Describing the metabolic profile during the early phase after SAH	Peak levels of CMD-glutamate, CMD-glucose, and the CMD-LPR occurred within the first 24 h of monitoring and decreased over time. CMD-pyruvate concentrations increased compared to baseline values. A higher CMD-LPR was associated with poor outcome. Higher CMD-IL-6 levels were associated with DCI and poor outcome
(15)	Single-center, prospective, observational	39	Hunt and Hess grade I + II *n* = 3 (8%), III *n* = 6 (15%), IV *n* = 12 (31%), V *n* = 18 (46%)	Data are reported for days 2–10 after SAH	Frontal, contralateral to the craniotomy in clipped patients; non-dominant hemisphere in diffuse SAH or ipsilateral in lateralized SAH in coiled patients	Comparing brain metabolism of patients with and without GCE on admission	Patients with GCE showed a higher CMD-LPR and lower CMD-pyruvate and CMD-glucose levels compared to those without. Episodes of CMD-LPR > 40 and metabolic crisis (CMD-LPR > 40 and CMD-glucose < 0.7 mmol/l) were more common in patients with GCE. CMD-LPR > 40 and metabolic crisis were associated with poor outcome
(16)	Single-center, prospective, observational	95	WFNS grade I *n* = 40 (42%), II *n* = 11 (11.5%), III *n* = 11 (11.5%), IV *n* = 20 (21%), V *n* = 13 (14%)	Monitoring was started 34/49 (mean) hours after SAH and maintained for 183/132 (mean) hours in patients with/without acute focal neurological deficits	Vascular territory of the aneurysm; insertion into lesioned tissue was avoided	Investigating brain metabolism in patients with/without acute focal neurological deficits	CMD-glutamate, CMD-glycerol, CMD-lactate concentrations, and the CMD-LPR were higher in patients with acute focal neurological deficits compared to those without. A normalization of values over time was concomitant with an improving clinical condition, further deterioration with permanent neurological deficits
(17)	Single-center, prospective, observational	97	WFNS grade I *n* = 37 (38%), II *n* = 13 (13%), III *n* = 9 (9%), IV *n* = 20 (21%), V *n* = 18 (19%)	Catheters were inserted within 72 h after SAH. Data are reported for days 1–10 after SAH	Vascular territory most likely affected by vasospasm; insertion into lesioned. tissue was avoided	Comparing brain metabolism of patients with acute neurological deficits and DCI to asymptomatic patients	In patients with acute focal neurological deficits, the CMD-glucose concentration was lower, whereas the CMD-lactate, CMD-LPR, CMD-glutamate and CMD-glycerol levels were significantly elevated compared to asymptomatic and DCI patients
(18)	Single-center, prospective, observational	149	WFNS grade 0 *n* = 3 (2%), I *n* = 53 (36%), II *n* = 16 (11%), III *n* = 17 (11%), IV *n* = 33 (22%), V *n* = 27 (18%)	Monitoring was started after aneurysm treatment (mean 24.7 h after SAH) and maintained for 161.8 h (mean)	Vascular territory of the aneurysm; insertion into lesioned tissue was avoided	Investigating the relationship between clinical disease severity, brain metabolism and outcome	The concentrations of all parameters were higher in high-grade (WFNS IV–V) compared to low-grade patients, the differences were significant for CMD-lactate, CMD-LPR and, during the first 2 days, CMD-glycerol
(19)	Single-center, prospective, observational	36	All patients had a WFNS grade of IV or V	Surgery was performed 44/30.7 h after SAH in patients with/without intracranial hypertension. Only patients with complete datasets for the first 7 days were included	Vascular territory of the aneurysm; insertion into lesioned tissue was avoided	To elucidate the impact of intracranial hypertension on brain metabolism	Patients with intracranial hypertension (ICP > 20 mmHg) had significantly lower levels of CMD-glucose and a higher CMD-LPR over the first 7 days after SAH. CMD-glutamate levels were significantly elevated in patients with high ICP on day 1
(20)	Single-center, prospective, observational	26	Hunt and Hess grade II *n* = 1 (4%), III *n* = 7 (26%), IV *n* = 2 (8%), V *n* = 16 (62%)	Monitoring was started 1 day (median) after SAH and maintained for 4 days (median)	Vascular territory of the aneurysm; classified as normal-appearing or perilesional brain tissue	To investigate the association between neuroinflammation, axonal injury and brain metabolism	High-grade neuroinflammation (CMD-IL-6 levels above median) was associated with CMD-lactate levels > 4 mmol/l, metabolic distress (CMD-LPR > 40), metabolic crisis (CMD-LPR > 40 and CMD-glucose levels < 0.7 mmol/l), DCI and poor functional outcome
(21)	Single-center, prospective, observational	52	WFNS grade I *n* = 4 (8%), II *n* = 11 (21%), III *n* = 2 (4%), IV *n* = 24 (46%), V *n* = 11 (21%)	Monitoring was started 20/28 h (mean) after SAH and maintained for 147/136 h (mean) in patients with/without GCE	Frontal, location in non-injured brain tissue; in 6 patients the CMD probe was located at the craniotomy site	Comparing brain metabolism of patients with and without global cerebral edema on admission	CMD-lactate and CMD-pyruvate levels were significantly, the CMD-LPR non-significantly higher in patients with GCE. There was no difference in CMD-glucose concentrations
(22)	Single-center, prospective, observational	19	Level of consciousness according to the Reaction Level Scale 85 on admission, conscious *n* = 11 (58%), unconscious *n* = 8 (42%)	Monitoring was started 21 h (median) after SAH and maintained for 157 h (median)	Cortical, frontal, in non-injured brain tissue; in 3 patients the CMD probe was located at the craniotomy site	Investigating the association between cerebral metabolites and the level of consciousness on admission	Patients who were unconscious on admission had significantly lower levels of CMD-pyruvate between 96 and 132 h after SAH

*SAH, subarachnoid hemorrhage; CMD, cerebral microdialysis; LPR, lactate-to-pyruvate-ratio; IL-6, interleukin-6; GCE, global cerebral edema; WFNS, world federation of neurological societies; DCI, delayed cerebral ischemia; ICP, intracranial pressure*.

**Table 4:** The clinical use of CMD as a marker of cerebral hypoperfuison and DCI in SAH patients.

Reference	Study type	Number of patients with SAH	Patient characteristics	Monitoring period	Probe location	Definition of ischemia/DCI	Study aim	Main microdialysis findings
(23)	Single-center, prospective, observational	19	Hunt and Hess grade II *n* = 2 (10%), III *n* = 3 (16%), IV *n* = 10 (53%), V *n* = 4 (21%)	Monitoring was started 1.4 days (mean) after SAH and was maintained for 5.8 days (mean)	Normal-appearing white matter between anterior and middle cerebral artery territory on the side of maximal pathology	Neurological worsening (GCS and NIHSS), persistent P_bt_O_2_ < 15 mmHg, flow velocity > 180 cm/s (transcranial Doppler), decreased alpha variability on continuous EEG or reduced blood flow on CT perfusion	To assess CMD-LPR levels with respect to established thresholds of CPP and P_bt_O_2_	The CMD-LPR was higher and episodes of CMD-LPR > 40 occurred more often when the CPP was < 60 mmHg. Brain tissue hypoxia was associated with CMD-LPR > 40. About 50% of P_bt_O_2_ measurements and 80% of CPP measurements were within normal range when the CMD-LPR was > 40. An LPR > 40 was associated with hospital mortality
(24)	Single-center, prospective, observational	16	Hunt and Hess grade II *n* = 13 (81%), III (19%)	All patients were operated within 1–3 days after SAH	Cortical, frontal or temporal, ipsilateral to the aneurysm	Vasospasm-related clinical disturbances	Associating CMD findings with impending ischemia	Patients with DCI showed increasing levels of CMD-lactate and decreasing levels of CMD-glucose. CMD-glutamate levels were increased in the proximity of infarcts on head CT
(25)	Single-center, prospective, observational	6	Hunt and Hess grade II *n* = 2 (33.3%), III *n* = 2 (33.3%), IV *n* = 2 (33.3%)	n/a	Cortical, frontal	By PET: regional oxygen extraction ratio > 125% and CMRO_2_ ≥ 45% of the corresponding contralateral region or CMRO_2_ < 45% of the corresponding contralateral region	To associate brain metabolite concentrations with ischemia detected by PET	Ischemia, defined using the cerebral metabolic rate of oxygen and oxygen extraction ratio detected by PET, was concomitant with high levels of CMD-lactate, CMD-glutamate, and CMD-LPR. No statistical analysis was performed
(26)	Single-center, prospective observational	32	Hunt and Hess grade I + II *n* = 3 (9%), III *n* = 6 (19%), IV *n* = 9 (28%), V *n* = 14 (44%)	CMD data are reported in relation to head CT scans between 1 and 10 days after the hemorrhage	White matter; frontal, contralateral to the craniotomy in clipped patients; non-dominant hemisphere in diffuse SAH or ipsilateral in lateralized SAH in coiled patients	Clinical symptoms or new infarcts on CT or MRI attributable to vasospasm	Comparing the metabolic patterns preceding head CT scans with and without new infarcts	CMD-lactate levels and the CMD-LPR significantly increased and CMD-glucose concentrations significantly decreased (to 0.5 mmol/l) when new infarcts were detected on a head CT scan. This was not observed in contralateral (from the CMD probe) or distant (>4 cm) infarction. Metabolic crisis (CMD-LPR > 40 and CMD-glucose < 0.7 mmol/l) was more common when new infarcts were revealed
(27)	Single-center, prospective observational	4	Hunt and Hess grade II *n* = 2 (50%), III *n* = 1 (25%), IV *n* = 1 (25%)	CMD was started between 12 and 28 h after SAH and maintained until about 200 h after SAH	Cortical, frontal	Neurological signs and neuro-imaging (CT/PET)	Associating the temporal dynamics of cerebral CMD-glycerol levels with ischemic events	Ischemic events were associated with a pronounced increase in CMD-glycerol levels (descriptive). In 1 patient without ischemia CMD-glycerol remained low after the peak immediately at the start of monitoring. CMD-glycerol levels correlated with CMD-LPR, CMD-glutamate, and CMD-lactate concentrations
(28)	Single-center, prospective, observational	55	Admission GCS 15 *n* = 9 (16%), 9–14 *n* = 20 (36%), <9 *n* = 26 (47%)	n/a	Craniotomy site in patients undergoing open surgery; frontal in patients not undergoing open surgery	Biochemical: CMD-LPR > 30 and CMD-pyruvate < 70 μmol/l	Proposing a metabolic pattern suggestive for mitochondrial dysfunction	The pattern of mitochondrial dysfunction (LPR > 30 and pyruvate > 70 μmol/l) was more common (7.5-fold) than the pattern of ischemia (LPR > 30 and pyruvate < 70 μmol/l) and associated with higher levels of glucose and lower levels of glutamate and glycerol
(29)	Single-center, prospective, observational	170	WFNS grade 0 *n* = 3 (2%), I *n* = 58 (34%), II *n* = 22 (13%), III *n* = 18 (11%), IV *n* = 38 (22%), V *n* = 31 (18%)	Data are reported for days 1–7 after SAH	Vascular territory of the aneurysm; insertion into lesioned tissue was avoided	Symptomatic vasospasm	To compare systemic and CMD-glucose levels with respect to acute focal neurological deficits and DCI	Patients with acute neurological deficits and patients developing DCI had higher blood glucose levels on admission and over the first 7 days compared to asymptomatic patients, but significantly lower CMD-glucose levels. The CMD-LPR was highest in patients with acute neurological deficits, followed by DCI patients and asymptomatic patients
(30)	Single-center, prospective observational	10	Hunt and Hess grade II *n* = 2 (20%), III *n* = 7 (70%), IV *n* = 1 (10%)	Catheters were inserted 1 day (median) after SAH, CMD was performed for 4–11 days (median)	Cortical, frontal or temporal, ipsilateral to the aneurysm	Clinical deterioration associated with cerebral vasospasm	Introducing a bedside analyzer. Associating metabolic patterns with ischemia	DCI was associated with high levels of CMD-lactate, CMD-glutamate, CMD-glycerol, and CMD-LPR and low concentrations of CMD-glucose. Assumed “normal” ranges of metabolites: 1–4 mmol/l (CMD-glucose), 1–3 mmol/l (CMD-lactate), 10–50 µmol/l (CMD-glycerol), 2–10 µmol/l (CMD-glutamate), 10–40 (CMD-LPR)
(31)	Single-center, retrospective, observational	21	Modified Fisher scale III *n* = 6 (29%), IV *n* = 15 (71%)	Time point of monitoring start not described. Monitoring lasted 10 days in all patients	Frontal, ipsilateral to the most prominent pathology, intact brain tissue	n/a	Correlating the concentrations of cerebral metabolites with CBF	There was a positive correlation of CBF (measured by a thermo-dilution probe) with CMD-pyruvate and CMD-glucose levels and a negative correlation with CMD-lactate and CMD-glycerol levels and the CMD-LPR
(32)	Single-center, prospective observational	20	WFNS grade II *n* = 6 (30%), IV *n* = 2 (10%), V *n* = 12 (60%)	CMD sampling was started 14 h (median) after SAH and maintained for 8 days (median)	White matter, frontal watershed of the non-dominant hemisphere, visually normal brain	CBF < 32.5 ml/100 g/min and mean transit time > 5.7 s in CT perfusion	Comparing metabolic profiles between patients with and without cerebral hypoperfusion measured by CT	The critical perfusion threshold was defined as CBF < 32.5 ml/100 g/min and mean transit time > 5.7 s. Patients with hypoperfusion had a significantly higher CMD-LPR (51 vs. 31) and lower CMD-glucose levels (0.64 vs. 1.22 mmol/l). During the 18 h before the perfusion CT was performed, there was a significant increase in CMD-LPR and decrease in CMD-glucose levels in the hypoperfusion group, but not in patients with normal CBF
(33)	Single-center, prospective observational	10	Hunt and Hess grade II *n* = 3 (30%), III *n* = 4 (40%), IV *n* = 3 (30%)	Start time is not exactly given (figures indicate 12–30 h after SAH). Monitoring lasted 6–11 days (range)	Cortical, frontal	CT findings and clinical course	To match CMD data with CT findings, clinical course and outcome	CMD-lactate elevations were frequently observed without obvious cause, while CMD-LPR reflected ischemia and, during days 0–4, correlated with outcome. In an infracted area, CMD-glucose levels fell to and remained at 0. Zero-levels of CMD-glucose were observed more frequently in patients with poor outcome. Patients with poor outcome had significantly higher CMD-glutamate levels
(34)	Single-center, prospective, observational	18	WFNS grade I *n* = 4 (22%), II *n* = 4 (22%), III *n* = 1 (6%), IV *n* = 6 (33%), V *n* = 2 (17%)	CMD monitoring started within 24 h after admission. Data were analyzed on days 1–12 after SAH	Vascular territory of the aneurysm	New focal neurological impairment or decrease ≥ 2 points in GCS score for at least 1 h, not attributable to other causes	Investigating an association between early onset pneumonia and cerebral metabolism	Elevated lactate levels on day 7 were associated with DCI
(35)	Single-center, prospective, observational	9	Hunt and Hess grade I *n* = 2 (22.2%), II *n* = 2 (22.2%), III *n* = 2 (22.2%), IV *n* = 2 (22.2%), V *n* = 1 (11.1%)	Within 72 h of admission	Cortical, right frontal lobe	Neurologic deficit or deterioration that could not be explained by other reasons	Associating the concentrations of cerebral metabolites with CBF	Lower CBF, measured by Xenon-CT, occurred together with higher levels of CMD-glutamate and a higher CMD-LPR. There was a descriptive association between CMD-LPR > 25 and a CBF < 22 ml/100 g/min. No statistical analyses were performed
(36)	Single-center, prospective, observational	78	WFNS grade I *n* = 26 (33%), II *n* = 11 (14), III *n* = 9 (12%), IV *n* = 18 (23%), V *n* = 14 (18%)	Monitoring was started 46 h (mean) after SAH and maintained for 155 h (mean)	White matter, vascular territory most likely affected by vasospasm	Insidious onset of confusion or appearance of a focal neurological deficit	To assess the sensitivity and specificity of CMD for confirming DCI	Baseline values did not differ between patients with and without DCI. Threshold values were set at CMD-lactate > 4 mmol/l and CMD-glutamate > 3 μmol/l. CMD showed a higher specificity for confirming DCI than conventional angiography and TCD
(37)	Single-center, prospective observational	33	WFNS grade 3.5 (median), 1–5 (range)	Monitoring was started 29.5 h (mean) after SAH and maintained for 112 h (mean)	Cortical, frontal or temporal, visually non-injured tissue	Decrease in the level of consciousness (≥1 step in the RLS score) or new focal neurological deficit, not due to other causes but vasospasm	Identifying a metabolic pattern indicative of ischemia	Five hours of CMD-LPR > 40 during a 10-h period were defined as ischemic pattern. 12 episodes of this pattern occurred, of which 5 were attributable to early infarcts and 6 to DCI. Only 6 of 15 cases of DCI were associated with this pattern, which, in these cases, occurred 16.7 h before DCI
(38)	Single-center, prospective, observational	7	Symptoms on admission, 3 patients (43%) were described as asymptomatic, 4 (57%) suffered from either aphasia or hemiparesis	Clipping and CMD probe insertion were performed within 24 h after SAH. The mean monitoring time was 8.5 days	Vascular territory of the aneurysm; insertion in lesioned tissue was avoided	New focal neurological signs or deterioration in level of consciousness, excluding other causes but vasospasm	To associate PET findings indicative of hypoxia with CBF and cerebral metabolism	In regions with ^18^F-FMISO uptake, a PET marker of hypoxia, CMD-glutamate levels were significantly higher compared to regions without uptake. No differences in energy metabolite concentrations were observed
(39)	Single-center, prospective observational	15	Neurological symptoms, 5 patients (33.3%) were classified as asymptomatic. 10 patients (66.6%) suffered either from aphasia (1), frontal lobe dysfunction (2), paresis (5) or coma (2)	Monitoring was started 52.8 h (mean) after SAH and maintained for 201/211 h (mean) in patients with/without symptoms of ischemia	Brain parenchyma most likely affected by vasospasm	Neurological deficits	To associate brain metabolite concentrations with CBF and ischemia measured by PET	On the day of PET, levels of CMD-lactate, CMD-glutamate, CMD-glycerol and the CMD-LPR were significantly higher in symptomatic patients. There were strong inverse correlations between CBF (measured by PET) and CMD-glutamate and CMD-glycerol levels
(40)	Single-center, prospective observational	13	WFNS grade I *n* = 3 (23%), II *n* = 2 (15.5%), III *n* = 4 (33%), IV *n* = 3 (23%), V *n* = 1 (15.5%)	Monitoring was started 52.8 h (mean) after SAH and maintained for 201 h (mean)	White matter, vascular territory most likely affected by vasospasm; insertion in lesioned tissue was avoided	Worsening of headache or focal neurological deficits not present at admission, between 2 and 14 days after SAH, not attributable to other causes	Associating CMD parameters with symptoms of ischemia and CBF	3-day medians were compared between symptomatic (ischemic) and asymptomatic intervals. The CMD-LPR, CMD-lactate, and CMD-glutamate levels were higher during symptomatic intervals. There were strong inverse correlations between CBF (measured by PET) and CMD-glutamate and CMD-glycerol levels. CMD-lactate levels > 4 mmol/l were an indicator of critically low CBF (< 20 ml/100 g/min)
(17)	Single-center, prospective, observational	97	WFNS grade I *n* = 37 (38%), II *n* = 13 (13%), III *n* = 9 (9%), IV *n* = 20 (21%), V *n* = 18 (19%)	Catheters were inserted within 72 h after SAH. Data are reported for days 1–10 after SAH	Vascular territory most likely affected by vasospasm; insertion into lesioned tissue was avoided	Worsening of headache, stiff neck, insidious onset of confusion, disorientation or drowsiness, or focal neurological deficits, between 2 and 14 days after SAH, not attributable to other causes	Comparing brain metabolism of patients with acute neurological deficits and DCI to asymptomatic patients	DCI patients had higher lactate and glutamate concentrations on days 1–8 and a higher LPR on days 3–8 compared with asymptomatic patients
(41)	Single-center, prospective, observational	30	Hunt and Hess grade II + III *n* = 5 (17%), IV *n* = 10 (33%), V *n* = 15 (50%)	Monitoring was started 3 days (median) after SAH and maintained for 110 h (median)	Frontal, ipsilateral to lateralized aneurysms, right frontal lobe in case of midline aneurysms	Clinical deterioration or cerebral infarction attributable to vasospasm	To identify the relation between CPP thresholds and brain metabolic crisis	Metabolic crisis (CMD-LPR > 40 and CMD-glucose levels ≤ 0.7 mmol/l) was associated with a CPP < 70 mmHg, Hunt and Hess grade 5, intraventricular or parenchymal hemorrhage, hydrocephalus, ICP > 20 mmHg and serum glucose levels < 6.6 mmol/l. Metabolic crisis was associated with poor outcome
(42)	Single-center, prospective observational	18	Hunt and Hess grade I *n* = 4 (22%), II *n* = 4 (22%), III *n* = 7 (39%), IV *n* = 3 (17%)	Time point of monitoring start not described. Monitoring was maintained up to 7 days after SAH	Subcortical, either radiologically normal-appearing brain tissue or ischemic tissue indicated by brain CT	Infarction on cerebral CT scans	Comparing brain metabolism between patients without ischemia and patients suffering brain death	In patients without evidence of cerebral ischemia CMD-glucose and CMD-pyruvate levels were significantly higher and CMD-glutamate, CMD-glycerol, and CMD-lactate levels and the CMD-LPR were lower compared to patients becoming brain dead. During the time between brain death (complete ischemia) and cessation of treatment, CMD-glucose, and CMD-pyruvate were not detectable and there was a further increase of CMD-glutamate and CMD-glycerol levels
(43)	Single-center, prospective observational	42	WFNS grade I *n* = 13 (31%), II *n* = 10 (24%), III *n* = 3 (7%), IV *n* = 13 (31%), V *n* = 3 (7%)	Time point of monitoring start not described. CMD was performed for 5 days (mean)	Cortical and white matter, vascular territory of the parent vessel of the aneurysm	Diagnosed by the neurosurgeon on call	Assessing the predictive value of a CMD pattern for DCI	The pattern was defined as an increase in CMD-LPR and lactate-to-glucose-ratio > 20%, followed by an increase in CMD-glycerol levels > 20%. In 17 of 18 patients, in whom DCI occurred, the pattern was found. It preceded the event by 11 h (glycerol peak to DCI). The ischemic pattern occurred in 3 patients without DCI
(44)	Single-center, prospective observational	60	WFNS grade I *n* = 20 (33%), II *n* = 9 (15%), III *n* = 5 (8%), IV *n* = 15 (25%), V *n* = 11 (18%)	Monitoring was started after clipping, 28 h (mean) after SAH. Monitoring was maintained for 174 h (mean)	White matter, vascular territory most likely affected by vasospasm; insertion into lesioned tissue was avoided	Symptomatic vasospasm defined as insidious onset of confusion or focal neurological deficit	To assess the predictive ability of CMD regarding DCI compared to TCD and conventional angiography	Baseline values (first 72 h) did not differ between DCI and non-DCI patients. In DCI patients, CMD-glucose levels decreased (64%) and CMD-lactate and CMD-glutamate levels increased (112 and 400%) thereafter. The pathological threshold was defined as CMD-lactate levels > 4 mmol/l and CMD-glutamate levels > 3 μmol/l for 6 consecutive hours. Using this pattern, CMD showed a higher specificity than TCD and angiography

*SAH, subarachnoid hemorrhage; CMD, cerebral microdialysis; LPR, lactate-to-pyruvate-ratio; CPP, cerebral perfusion pressure; P_bt_O_2_, brain tissue oxygen tension; n/a, data not available; PET, positron emission tomography; WFNS, world federation of neurological societies; DCI, delayed cerebral ischemia; CBF, cerebral blood flow; FMISO, fluoromisonidazole; TCD, transcranial Doppler; CT, computed tomography; GCS, Glasgow coma scale; EEG, electroencephalography; MRI, magnetic resonance imaging; NIHSS, National Institutes of Health Stroke Scale; CMRO_2_, cerebral metabolic rate of oxygen; RLS, reaction level scale*.

**Table 5:** CMD in monitoring treatment effects in SAH patients.

Reference	Study type	Number of patients with SAH	Patient characteristics	Monitoring period	Probe location	Study aim	Main microdialysis findings
(45)	Single-center, prospective, interventional	14	n/a	n/a	Frontal or parietal lobe ipsilateral to the aneurysm	To assess the impact of hypertonic saline on cerebral perfusion and metabolism	30 and 60 min after the infusion of hypertonic saline, the CMD-LPR decreased in 9 of 14 patients. Overall, this effect was not significant
(46)	Single-center, prospective, observational	9/12	Hunt and Hess grade 5 (median), 4–5 (interquartile range)	Monitoring was initiated at day 2 (median) after SAH and maintained for 8 days (median)	White matter, frontal, hemisphere deemed at greatest risk for secondary injury	To assess the impact of intravenous mannitol on cerebral metabolism	Mannitol was administered due to an ICP crisis > 20 mmHg. The highest CMD-LPR was recorded at the time point of the start of the infusion (mean of 47). The CMD-LPR significantly decreased by 20% over 2 h, CMD-lactate and CMD-pyruvate levels decreased non-significantly, CMD-glucose remained unaffected
(47)	Single-center, retrospective, observational	34	Hunt and Hess grade III *n* = 6 (18%), IV *n* = 12 (35%), V *n* = 16 (47%)	n/a	White matter, frontal, hemisphere deemed at greatest risk for secondary injury	Comparing the frequencies of metabolic distress between different hemoglobin levels	Compared to hemoglobin concentrations between 10 and 11 g/dl, episodes of CMD-LPR > 40 occurred 1.9 times more often when hemoglobin was between 9 and 10 g/dl, and 3.8 times more often when hemoglobin was below 9 g/dl (45% of measurements showed CMD-LPR > 40, respectively)
(48)	Single-center, prospective, observational	15	Hunt and Hess grade IV + V in 80% of patients	n/a	White matter, hemisphere deemed at greatest risk for secondary injury or right frontal lobe; visually normal tissue	Investigating the impact of packed red blood cell infusions on brain metabolism	Over a 12-h period after a packed red blood cell infusion, no significant changes in cerebral metabolism were observed, despite an increase in CPP and P_bt_O_2_
(49)	Single-center, prospective, observational	18	WFNS grade I *n* = 3 (17%), III *n* = 2 (11%), IV *n* = 4 (22%), V *n* = 9 (50%)	Catheters were inserted 15/12 h (median) after SAH and CMD was maintained for 164/180 h (median) in patients with/without craniectomy	Vascular territory of the aneurysm; insertion into lesioned tissue was avoided	To assess the impact of decompressive craniectomy (due to refractory intracranial hypertension) on brain metabolism	Compared to a control group with normal ICP, patients with intracranial hypertension (ICP < 20 mmHg for > 6 h) had lower levels of CMD-glucose and higher levels of CMD-lactate, CMD-glutamate, CMD-glycerol, and CMD-LPR. Concentrations of CMD-glucose and CMD-pyruvate were higher and levels of CMD-glycerol were lower in patients who underwent decompressive craniectomy compared to those who were treated conservatively. The metabolic pattern of CMD-LPR > 25, CMD-glycerol > 80 μmol/l and CMD-glutamate > 10 μmol/l for > 6 h preceded the onset of refractory intracranial hypertension by 40 h (median)
(50)	Single-center, prospective, observational	182	WFNS grade 0 *n* = 3 (2%), I *n* = 61 (37%), II *n* = 23 (14%), III *n* = 17 (10%), IV *n* = 35 (21%), V *n* = 25 (15%)	Monitoring was started immediately after aneurysm treatment (23/13 h after SAH, mean) and maintained for 169/172 h (mean) in patients with/without ICP intracranial hypertension	Vascular territory of the aneurysm; insertion into lesioned tissue was avoided	Investigating the impact of intracranial hypertension on cerebral metabolism	Higher CMD-LPR, CMD-glutamate, and CMD-glycerol levels and lower CMD-glucose levels were observed in patients with ICP > 20 mmHg. A metabolic pattern of LPR > 25, glutamate > 10 μmol/l and glycerol > 80 μmol/l preceded the first ICP increase > 20 mmHg. Decompressive craniectomy was associated with a decrease in CMD-glycerol and an increase in CMD-glutamate levels. Higher CMD-LPR and CMD-glutamate levels were associated with poor outcome
(51)	Single-center, prospective, observational	18	Hunt and Hess grade II *n* = 2 (11%), III *n* = 4 (22%), IV *n* = 8 (45%), V *n* = 4 (22%)	Monitoring was initiated 1 day (median) after SAH. Between 37 and 168 hourly samples were obtained per patient	White matter, contralateral to the maximal focal injury, normal-appearing tissue	To assess the impact of induced normothermia on cerebral metabolism	When normothermia (37°C) was induced due to refractory fever (≥38.3°C), it was associated with a decrease in CMD-LPR and fewer episodes of metabolic distress (CMD-LPR > 40). Patients with poor outcome had a higher CMD-LPR
(52)	Single-center, prospective, observational	20	Hunt and Hess grade II *n* = 2 (10%), III *n* = 4 (20%), IV *n* = 10 (50%), V *n* = 4 (20%)	Monitoring was started with 48 h after SAH and maintained for 7 days (median)	White matter, vascular territory most likely affected by vasospasm; radiologically normal-appearing tissue	To associate hemoglobin concentrations with cerebral metabolism	Hemoglobin concentrations < 9 g/dl were associated with a higher absolute CMD-LPR and more episodes of LPR > 40 compared to higher hemoglobin levels
(53)	Single-center, prospective, observational	33	WFNS grade 3 (median), 1–5 (range)	CMD sampling was started 29.5 h (mean) after SAH and maintained for 112 h (mean)	Cortical, frontal or temporal, non-injured brain tissue	To assess the relationship between ICP, CPP and cerebral metabolism	CPP was positively correlated with CMD-pyruvate levels. Episodes of ICP > 10 mmHg were associated with lower levels of CMD-pyruvate and higher levels of CMD-lactate, CMD-pyruvate, and CMD-LPR. In 3 patients, opening the ventricular drain was associated with increasing CMD-pyruvate levels (descriptive)
(54)	Single-center, prospective, observational	21	Hunt and Hess grade II + III *n* = 6 (29%), IV + V *n* = 13 (71%)	Monitoring was initiated at day 1 (median) after SAH and maintained for 12 (median) days	White matter, hemisphere deemed at greatest risk for secondary injury	To assess the impact of intravenous diclofenac on CPP, P_bt_O_2_ and cerebral metabolism	Despite a decrease of body temperature, CPP and P_bt_O_2_, no changes in cerebral metabolism were observed
(55)	Single-center, prospective, randomized, controlled, double-blind	54 (35 with CMD)	WFNS grade I *n* = 22 (41%), II *n* = 5 (9%), III *n* = 1 (2%), IV *n* = 16 (30%), V *n* = 10 (18%)	Data are reported for days 1–14 after SAH	Vascular territory of the aneurysm	To study the efficacy and safety of EPO in SAH patients	The administration of EPO was associated with higher CMD-glycerol levels. No differences in other CMD parameters were observed
(56)	Single-center, prospective, observational	11	Hunt and Hess grade III *n* = 3 (27%), IV *n* = 2 (18%), V *n* = 6 (55%)	Measurements were performed between after 4–14 days (range) after SAH	White matter, frontal watershed, ipsilateral to the aneurysm or contralateral to the craniotomy in clipped patients	To investigate the impact of intraarterial verapamil on brain metabolism	There was a significant increase in CMD-glucose levels 9 h after the administration of intraarterial verapamil (1.2–1.53 mmol/l). No significant changes in other CMD parameters were observed

*n/a, data not available; CMD, cerebral microdialysis; LPR, lactate-to-pyruvate-ratio; SAH, subarachnoid hemorrhage; CPP, cerebral perfusion pressure; P_bt_O_2_, brain tissue oxygen tension; ICP, intracranial pressure; EPO, erythropoietin*.

**Table 6:** CMD and systemic glucose management in SAH patients.

Reference	Study type	Number of patients with SAH	Patient characteristics	Monitoring period	Probe location	Study aim	Main microdialysis findings
(57)	Single ceter, prospective, observational	28	Hunt and Hess grade III *n* = 6 (21%), IV *n* = 8 (29%), V *n* = 14 (50%)	Monitoring was initiated on day 2 (median) after SAH and maintained for 6 days (median)	White matter, frontal, tissue at risk or contralateral to the craniotomy	To assess the relationship between rapid reductions in serum glucose and brain metabolism	Reductions in serum glucose by 25% (within normal range) were independently associated with consecutive metabolic crisis (CMD-LPR > 40 and CMD-glucose < 0.7) and increasing CMD-LPR. There was a concomitant decrease in CMD-glucose and CMD-pyruvate. A higher CMD-LPR was associated with hospital mortality
(58)	Single ceter, prospective, observational	12	Glasgow coma scale score 5–7 (range)	Measurements took place between 1 and 5 days after SAH	Cortical, right frontal lobe	To investigate the impact of enteral nutrition on cerebral metabolism	CMD-glucose levels significantly increased following the first bolus of enteral nutrition (2.5–3.7 mmol/l). No changes in CMD-lactate, CMD-pyruvate, CMD-glutamate, or CMD-glycerol were observed. No insulin was used during the measurements
(59)	Single ceter, prospective, observational	17	Hunt and Hess grade II *n* = 2 (12%), III *n* = 6 (35%), IV *n* = 2 (12%), V *n* = 7 (41%)	Measurements took place between 3 and 22 days after SAH	White matter, hemisphere deemed at greatest risk for secondary injury, classified as normal-appearing or perilesional brain tissue	To investigate the impact of enteral nutrition on cerebral glucose levels	Enteral nutrition significantly increased CMD-glucose levels (1.59–2.03 mmol/l) with a delay of 3 h. This increase was independent of insulin administration, absolute levels of serum glucose, evidence of cerebral metabolic distress, and microdialysis probe location. Also critically low CMD-glucose concentrations were increased. There was a significant association between serum and CMD-glucose levels
(60)	Single ceter, prospective, observational	28	Hunt and Hess grade II *n* = 1 (4%), III *n* = 5 (18%), IV *n* = 8 (29%), V *n* = 14 (50%)	Monitoring was started 2 days (median) after SAH and maintained for 6 days (median)	White matter, frontal, hemisphere deemed at greatest risk for secondary injury	To investigate the impact of blood glucose variability on cerebral metabolism	A higher systemic glucose variability, defined as the SD of measured concentrations per day, was associated with a higher risk of developing at least one episode of CMD-LPR > 40 per day
(61)	Single ceter, prospective, observational	10/20	GCS score 7 (median), 3–10 (range)	CMD monitoring was started 45 h (median) after SAH and maintained for 96 h (median)	Frontal, near the area of lesioned tissue or right frontal lobe in patients with diffuse injury	Investigating the impact of tight glycemic control on cerebral metabolism	Blood glucose levels were defined as “tight” (4.4–6.7 mmol/l) or “intermediate” (6.8–10 mmol/l). Tight blood glucose was associated with lower levels of CMD-glucose, more episodes of CMD-glucose < 0.7 mmol/l, higher CMD-LPR and a more frequent occurrence of metabolic crisis (CMD-LPR > 40 and CMD-glucose levels < 0.7 mmol/l). Low CMD-glucose levels and metabolic crisis were associated with higher hospital mortality
(62)	Single ceter, prospective, observational	178	WFNS grade I *n* = 65 (37%), II *n* = 22 (12%), III *n* = 20 (11%), IV *n* = 36 (20%), V *n* = 35 (20%)	CMD was performed on days 1–7 after SAH	Vascular territory of the aneurysm; insertion into lesioned tissue was avoided	To investigate the associations between hyperglycemia, cerebral metabolism and outcome	CMD-glucose levels were higher during serum glucose > 7.8 mmol/l. No differences in other microdialysis parameters were observed
(63)	Single ceter, prospective, observational	28	WFNS grade I *n* = 8 (29%), II *n* = 6 (21%), III *n* = 3 (11%), IV *n* = 6 (21%), V *n* = 5 (18%)	Monitoring was initiated 22.2 h (mean) after SAH and maintained for 195.4 h (mean)	Vascular territory of the aneurysm; insertion into lesioned tissue was avoided	To investigate the impact of hyperglycemia on cerebral metabolism	During episodes of blood glucose > 140 mg/dl, CMD-lactate and CMD-pyruvate concentrations increased, the CMD-LPR remained stable, and CMD-glutamate levels decreased. Serum glucose concentrations did not differ during episodes of high (>2.6 mmol/l) and low (<0.6 mmol/l) CMD-glucose concentrations. During episodes of low CMD-glucose, CMD-lactate, CMD-glutamate, CMD-glycerol levels, and the CMD-LPR were elevated. Low CMD-glucose during blood glucose > 140 mg/dl was associated with poor outcome
(64)	Single ceter, prospective, observational	31	WFNS grade I *n* = 9 (29%), II *n* = 6 (19%), III *n* = 3 (10%), IV *n* = 6 (19%), V *n* = 7 (23%)	The mean duration of monitoring was 192/295 h in patients with/without insulin. Data are reported for days 1–10 after SAH	Vascular territory of the aneurysm; insertion into lesioned tissue was avoided	To investigate the impact of intravenous insulin on cerebral metabolism	CMD-glucose levels, but not serum glucose levels, decreased significantly 3 h after the start of the insulin infusion. Episodes of low CMD-glucose (<0.6 mmol/l) were (non-significantly) more common in patients who received insulin. CMD-lactate and CMD-pyruvate levels did not change, CMD-glycerol concentrations slightly increased, and CMD-glutamate levels decreased after the start of insulin treatment
(65)	Single-center, prospective, observational	24	WFNS grade I *n* = 5 (21%), II *n* = 4 (17%), III *n* = 2 (8%), IV *n* = 6 (25%), V *n* = 7 (29%)	Data were collected on days 1–10 after SAH	Vascular territory of the aneurysm; insertion into lesioned tissue was avoided	To investigate the long-term effect of insulin on cerebral metabolism	Median daily CMD-glucose levels decreased after the initiation of insulin treatment and were significantly lower, compared to baseline, 4 days after insulin start. A significant increase in CMD-glycerol levels was observed 1 day after insulin start, which was not significant thereafter. CMD-glutamate levels significantly decreased over time
(66)	Single ceter, retrospective, observational	50	Hunt and Hess grade II *n* = 3 (6%), III *n* = 7 (14%), IV *n* = 15 (30%), V *n* = 25 (50%)	Monitoring was started 2 days (median) after SAH and maintained for 108 h (mean)	Normal-appearing white matter	To elucidate the relations between enteral nutrition, insulin treatment and cerebral metabolism	There was no direct association between CMD-glucose levels and the energy content of the administered enteral nutrition. There was a significant association between CMD and serum glucose levels. When the CMD-LPR was <40, higher CMD and serum glucose levels were associated with a higher insulin dose. When the CMD-LPR was >40, a higher insulin dose was associated with lower CMD-glucose levels, despite higher serum glucose concentrations
(67)	Single ceter, prospective, observational	19	WFNS grade I *n* = 1 (5.3%), II *n* = 1 (5.3%), III *n* = 1 (5.3%), IV *n* = 11 (58%), V *n* = 5 (26%)	The mean monitoring time was 147 h. Data are reported for days 1–7 after SAH	Cortical, frontal, classified as radiologically normal-appearing or adjacent to ischemic lesions	To elucidate the relation between brain and serum glucose levels	CMD-glucose levels decreased over days 1–7. There was a significant correlation between CMD and serum glucose levels (*r* = 0.27). CMD-lactate and CMD-pyruvate levels increased over time, beginning on day 3. CMD-glutamate levels peaked on day 1 and decreased thereafter. CMD-glucose and CMD-pyruvate levels decrease during insulin treatment, despite systemic glucose concentrations within normal range

*SAH, subarachnoid hemorrhage; CMD, cerebral microdialysis; LPR, lactate-to-pyruvate-ratio; GCS, Glasgow coma scale; WFNS, world federation of neurological societies*.

**Table 7:** Brain metabolism and outcome after SAH.

Reference	Study type	Number of patients with SAH	Patient characteristics	Monitoring period	Probe location	Study aim	Main microdialysis findings
(68)	Single-center, prospective, observational	28	Hunt and Hess grade II *n* = 3 (11%), III *n* = 6 (21%), IV *n* = 3 (11%), V *n* = 16 (57%)	Monitoring was initiated 1 day (median) after SAH. Data are reported up to 12 days after SAH	White matter at greatest risk for secondary brain injury; classified as normal or perilesional tissue	Investigating associations between CMD-K^+^ levels, brain metabolism and functional outcome	Elevated cerebral CMD-K^+^ levels (above the median of 3 mmol/l) were associated with CMD-LPR > 40, CMD-lactate > 4 mmol/l and poor outcome. CMD-K^+^ concentrations positively correlated with CMD-lactate and CMD-glutamate levels and the CMD-LPR. CMD-LPR > 40 was independently associated with poor functional outcome
(69)	Single-center, prospective, observational	20	Hunt and Hess grade III *n* = 10, IV *n* = 6, V *n* = 4	CMD monitoring started within 24 h after SAH in most patients and was maintained for 3–12 days (range)	Cortical, frontal	Describing metabolic profiles in relation to functional outcome	A metabolic pattern of decreasing CMD-glucose levels paralleled by an increase in both, CMD-lactate and CMD-pyruvate concentrations, after 24–72 h was common in patients with good outcome. A pattern of CMD-glucose levels remaining high combined with low CMD-pyruvate concentrations was common in patients with poor outcome
(70)	Single-center, prospective, randomized-controlled	30/60	GCS score average of 5	n/a	n/a	Comparing an ICP-based to a CPP-based treatment concept	Patients with poor outcome had significantly lower levels of CMD-glucose (1.1 vs. 2.1 mmol/l) and higher levels of CMD-glycerol and a higher CMD-LPR compared to patients with good outcome
(14)	Single-center, prospective, observational	26	Hunt and Hess grade II *n* = 2 (7.7%), III *n* = 6 (23.1%), IV *n* = 2 (7.7%), V *n* = 16 (61.5%)	Monitoring was started 22 h (median) after SAH and data of the following 144 h are reported	Frontal, ipsilateral to the aneurysm; classified as normal-appearing or perilesional brain tissue	Describing the metabolic profile during the early phase after SAH	A higher CMD-LPR was associated with poor outcome
(15)	Single-center, prospective, observational	39	Hunt and Hess grade I + II *n* = 3 (8%), III *n* = 6 (15%), IV *n* = 12 (31%), V *n* = 18 (46%)	Data are reported for days 2–10 after SAH	Frontal, contralateral to the craniotomy in clipped patients; non-dominant hemisphere in diffuse SAH or ipsilateral in lateralized SAH in coiled patients	Comparing brain metabolism of patients with and without global cerebral edema on admission	CMD-LPR > 40 and metabolic crisis (CMD-LPR > 40 and CMD-glucose < 0.7 mmol/l) were associated with poor outcome
(57)	Single ceter, prospective, observational	28	Hunt and Hess grade III *n* = 6 (21%), IV *n* = 8 (29%), V *n* = 14 (50%)	Monitoring was initiated on day 2 (median) after SAH and maintained for 6 days (median)	White matter, frontal, tissue at risk or contralateral to the craniotomy	To assess the relationship between rapid reductions in serum glucose and brain metabolism	A higher CMD-LPR was associated with hospital mortality
(71)	Single-center, prospective, observational	35/40	Admission WFNS grade IV + V *n* = 22	Data are reported for days 2–17 after SAH. The mean monitoring time per patient was 4 days	Frontal or suspected aneurysmal vascular territory	Investigating the association between brain metabolism and patient outcome	Patients with unfavorable outcome showed a higher CMD-LPR compared to those with good outcome. Episodes of CMD-LPR > 40 or CMD-glutamate levels > 10 μmol/l were both associated with cerebral infarction and poor outcome
(50)	Single-center, prospective, observational	182	WFNS grade 0 *n* = 3 (2%), I *n* = 61 (37%), II *n* = 23 (14%), III *n* = 17 (10%), IV *n* = 35 (21%), V *n* = 25 (15%)	Monitoring was started 23/13 h (mean) after SAH and maintained for 169/172 h (mean) in patients with/without intracranial hypertension	Vascular territory of the aneurysm; insertion into lesioned tissue was avoided	Investigating the impact of intracranial hypertension on cerebral metabolism	Higher CMD-LPR and CMD-glutamate levels were associated with poor outcome
(61)	Single ceter, prospective, observational	10/20	GCS score 7 (median), 3–10 (range)	CMD monitoring was started 45 h (median) after SAH and maintained for 96 h (median)	Frontal, near the area of lesioned tissue or right frontal lobe in patients with diffuse injury	Investigating the impact of tight glycemic control on cerebral metabolism	Blood glucose levels were defined as “tight” (4.4–6.7 mmol/l) or “intermediate” (6.8–10 mmol/l). Tight blood glucose was associated with lower levels of CMD-glucose, more episodes of CMD-glucose < 0.7 mmol/l, higher CMD-LPR and a more frequent occurrence of metabolic crisis (CMD-LPR > 40 and CMD-glucose levels < 0.7 mmol/l). Low CMD-glucose levels and metabolic crisis were associated with higher hospital mortality
(51)	Single-center, prospective, observational	18	Hunt and Hess grade II *n* = 2, III *n* = 4, IV *n* = 8, V *n* = 4	Monitoring was initiated 1 day (median) after SAH. Between 37 and 168 hourly samples were obtained per patient.	White matter, contralateral to the maximal focal injury, normal-appearing tissue	To assess the impact of induced normothermia on cerebral metabolism	When normothermia (37°C) was induced due to refractory fever (≥38.3°C), it was associated with a decrease in CMD-LPR and fewer episodes of metabolic distress (CMD-LPR > 40). Patients with poor outcome had a higher CMD-LPR
(72)	Two-center, prospective, observational	31	Comatose patients	Monitoring was started 1 day (median) after SAH and maintained for 7 days (median)	Visually normal white matter	To elucidate the relevance of elevated CMD-lactate levels in the context of brain tissue hypoxia and hyperglycolysis	Elevated CMD-lactate concentrations (>4 mmol/l) were defined as “hypoxic” (together with P_bt_O_2_ < 20 mmHg), “hyperglycolytic” (together with CMD-pyruvate concentrations > 119 μmol/l), or neither. Hyperglycolytic elevated CMD-lactate was more common than hypoxic elevated CMD-lactate and associated with favorable outcome. Hypoxic elevated CMD-lactate was associated with an increased mortality
(73) (abstract only)	Single-center, prospective, observational	18/51	n/a	n/a	n/a	To correlate CMD findings with functional outcome	Poor 12-month outcome was correlated with lower CMD-glucose levels and higher levels of CMD-glycerol and CMD-LPR
(33)	Single-center, prospective observational	10	Hunt and Hess grade II *n* = 3 (30%), III *n* = 4 (40%), IV *n* = 3 (30%)	Start time is not exactly given (figures indicate 12–30 h after SAH). Monitoring lasted 6–11 days (range)	Cortical, frontal	To match CMD data with CT findings, clinical course and outcome	CMD-lactate elevations were frequently observed without obvious cause, while CMD-LPR reflected ischemia and, during days 0–4, correlated with outcome. In an infracted area, CMD-glucose levels fell to and remained at 0. Zero-levels of CMD-glucose were observed more frequently in patients with poor outcome. Patients with poor outcome had significantly higher CMD-glutamate levels
(18)	Single-center, prospective, observational	149	WFNS grade 0 *n* = 3 (2%), I *n* = 53 (36%), II *n* = 16 (11%), III *n* = 17 (11%), IV *n* = 33 (22%), V *n* = 27 (18%)	Monitoring was started after aneurysm treatment (mean 24.7 h after SAH) and maintained for 161.8 h (mean)	Vascular territory of the aneurysm; insertion into lesioned tissue was avoided	Investigating the relationship between clinical disease severity, brain metabolism and outcome	Higher CMD-glutamate levels and CMD-LPR were independently associated with poor functional outcome and mortality
(20)	Single-center, prospective, observational	26	Hunt and Hess grade II *n* = 1 (4%), III *n* = 7 (26%), IV *n* = 2 (8%), V *n* = 16 (62%)	Monitoring was started 1 day (median) after SAH and maintained for 4 days (median)	Vascular territory of the aneurysm; classified as normal-appearing or perilesional brain tissue	To investigate the association between neuroinflammation, axonal injury and brain metabolism	High-grade neuroinflammation (CMD-IL-6 levels above median) was associated with poor functional outcome
(63)	Single ceter, prospective, observational	28	WFNS grade I *n* = 8 (29%), II *n* = 6 (21%), III *n* = 3 (11%), IV *n* = 6 (21%), V *n* = 5 (18%)	Monitoring was initiated 22.2 h (mean) after SAH and maintained for 195.4 h (mean)	Vascular territory of the aneurysm; insertion into lesioned tissue was avoided	To investigate the impact of hyperglycemia on cerebral metabolism	Low CMD-glucose levels during episodes of blood glucose concentrations > 140 mg/dl was associated with poor outcome
(41)	Single-center, prospective, observational	30	Hunt and Hess grade II + III *n* = 5 (17%), IV *n* = 10 (33%), V *n* = 15 (50%)	Monitoring was started 3 days (median) after SAH and maintained for 110 h (median)	Frontal, ipsilateral to lateralized aneurysms, right frontal lobe in case of midline aneurysms	To identify the relation between CPP thresholds and brain metabolic crisis	Metabolic crisis (CMD-LPR > 40 and CMD-glucose concentrations ≤0.7 mmol/l) was associated with poor outcome
(74)	Single-center, prospective, observational	10	Hunt and Hess grade I *n* = 2, II *n* = 3, III *n* = 1, IV *n* = 2, V *n* = 2	CMD was performed for 1.7–7 days (range). Data are reported up to 9 days after SAH	Frontal	Investigating associations between cerebral metabolism and patient outcome	Higher concentrations of CMD-glutamate and CMD-lactate were associated with poor functional outcome. In patients with poor outcome, glutamate levels followed a biphasic course with peaks on days 1–2 and 7. In patients with good outcome, glutamate levels remained low without any temporal dynamic
(75)	Single-center, prospective, observational	18	WFNS grade IV *n* = 4 (22%), V *n* = 14 (78%)	The median monitoring time was 8 days	Cortical, vascular territory of the aneurysm	Comparing CMD values to the arterio-jugular difference	No significant differences in absolute values of CMD parameters were observed between patients with good and poor outcome. CMD-lactate levels >4 mmol/l and CMD-pyruvate levels >119 μmol/l were significantly more common in patients with good outcome. Hypoxic elevated lactate (P_bt_O_2_ < 20 mmHg) was more common in patients with poor outcome

*SAH, subarachnoid hemorrhage; CMD, cerebral microdialysis; LPR, lactate-to-pyruvate-ratio; GCS, Glasgow coma scale; n/a, data not available; ICP, intracranial pressure; CPP, cerebral perfusion pressure; P_bt_O_2_, brain tissue oxygen tension; CBF, cerebral blood flow; IL-6, interleukin-6*.

**Table 8:** CMD findings not directly related to the discussed topics.

Reference	Study type	Number of patients with SAH	Patient characteristics	Monitoring period	Probe location	Study aim	Main microdialysis findings
(76) (abstract only)	n/a	n/a	n/a	Data are reported between 2 and 12 days after SAH	n/a	Investigating the effect of remote ischemic preconditioning on brain metabolism	Over the duration of remote ischemic preconditioning, the CMD-LPR and CMD-glycerol levels decreased. The effect persisted for 25–54 h
(77)	Single-center, prospective, observational	5	WFNS grade I *n* = 5 (100%)	All patients were operated within 1–3 days after SAH	Frontal or temporal, vascular territory most likely affected by vasospasm	Describing CMD levels in good-grade SAH patients	Mean levels of CMD parameters in WFNS grade I patients were: CMD-glucose 2.6 mmol/l, CMD-lactate 2.9 mmol/l, CMD-pyruvate 92 µmol/l, CMD-LPR 36, CMD-glutamate 11.2 µmol/l
(78)	Single-center, prospective, observational	17	WFNS grade I *n* = 1, II *n* = 3, III *n* = 5, IV *n* = 3, V *n* = 5	Probes were inserted within 72 h after SAH. Monitoring was performed for 85–336 h (range)	Vascular territory of the aneurysm; insertion into lesioned tissue was avoided	Investigating the association between CSD and brain metabolism	Patients with acute focal neurological deficits had higher baseline CMD-glutamate and CMD-lactate levels compared to those without. In patients without acute focal deficits, there was a significant transient decrease in CMD-glucose (−25%) and increase in CMD-lactate concentrations (+10%) following clusters of CSDs (2 or more per hour), but not single CSDs. No changes in CMD-glutamate levels were observed
(79) (abstract only)	Bi-center, prospective, observational	21	WFNS grade I–III *n* = 11, IV–V *n* = 10	Data are reported up to 14 days after SAH	Vascular territory at risk for DCI	To investigate an association between CSD and CMD-glucose level	CSD were not associated with changes in CMD-glucose levels
(80)	Single-center, retrospective, observational	167	WFNS grade 0 *n* = 3 (2%), I *n* = 58 (35%), II *n* = 21 (13%), III *n* = 18 (11%), IV *n* = 38 (23%), V *n* = 29 (17%)	Data are reported for days 1–10 after SAH. CMD was performed for 7–10 days	Vascular territory of the aneurysm; patients were excluded if the tip of the CMD probe was close to a parenchymal hemorrhage	To relate changes in cerebral metabolism to the emergence of bacterial meningitis	On the day when bacterial meningitis was diagnosed, CMD-glucose levels and the CMD-lactate-to-glucose-ratio were significantly lower than 3 days before. Compared to controls, only the decrease in CMD-glucose levels was more pronounced in patients with bacterial meningitis. A decrease in CMD-glucose levels of 1 mmol/l showed the highest combined sensitivity and specificity
(81) (Abstract only)	Single-center, prospective, observational	4	n/a	n/a	n/a	Investigating the impact of temperature changes on CMD-glutamate levels	In all patients, mild head cooling resulted in a significant decrease in CMD-glutamate levels. In 2 patients, CMD-glutamate concentrations increased sharply with fever
(82)	Single-center, prospective, observational	6/18	GCS motor score 3 *n* = 3, 5 *n* = 1, 6 *n* = 2	n/a	Cortical, normal-appearing tissue	To assess the impact of fever on cerebral metabolism	Neither the onset of fever (≥38.7°C) nor its resolution was associated with significant changes in cerebral metabolism

*n/a, data not available; SAH, subarachnoid hemorrhage; CMD, cerebral microdialysis; LPR, lactate-to-pyruvate-ratio; WFNS, world federation of neurological societies; CSD, cortical spreading depolarizations; DCI, delayed cerebral ischemia*.

### Post-Procedural Monitoring

We identified nine studies focusing on the early phase after aneurysm treatment (Table 3) (14–22). In summary, CMD-LPR > 40 during the early phase is a sensitive marker for poor clinical grade on admission (18), radiological evidence of global cerebral edema (15), intracranial hypertension (19), and poor 3-month outcome (14). Critically, low levels of CMD-glucose were associated with acute neurological deficits (16). Trend analysis may indicate the clinical course with “normalization of CMD-parameters” being associated with clinical improvement and pathological evolution of brain metabolism with permanent neurological deficits (16).

### CMD and Acute Focal Neurological Deficits after SAH

Most patients underwent aneurysm clipping. In a study including 26 poor-grade SAH patients (68% Hunt and Hess grade IV–V), CMD-LPR and CMD-glutamate were highest at the start of neuromonitoring, indicating metabolic distress (mean CMD-LPR > 40) and excitotoxicity, and significantly decreased thereafter (14). A higher CMD-LPR was associated with poor 3-month outcome (modified Rankin scale 4–6). CMD-glucose significantly decreased, however, did not reach critically low levels in this cohort with systemic glucose levels of 135–150 mg/dl (7.5–8.3 mmol/l) (14). A study including 149 SAH patients, of whom 89 (60%) were admitted with good clinical grades (WFNS grades ≤ 3), reported higher CMD-LPR and CMD-lactate levels in poor-grade patients, already at the start of neuromonitoring, compared to good-grade patients (18). Moreover, higher CMD-glycerol levels were reported in poor-grade patients, significantly decreasing over the first days (18).

In another study including 97 SAH patients, the authors compared patients with and without acute focal neurological deficits immediately after SAH due to SAH-related parenchymal hematoma and/or perioperative/periinterventional ischemia. In patients with focal deficits, CMD-lactate, CMD-LPR, CMD-glutamate, and CMD-glycerol were pathologically increased throughout the first week, whereas these parameters remained within normal range in patients without acute neurological deficits (16, 17). CMD-glucose levels were significantly lower in patients with acute focal deficits (16, 17), despite higher blood glucose concentrations (overall mean blood glucose levels were approximately 7.2–8.3 mmol/l = 130–150 mg/dl) (29). Regarding temporal dynamics, a trend toward normalization of CMD values was associated with clinical improvement, whereas further deterioration was associated with permanent neurological deficits in patients with acute focal deficits (16).

### CMD and Admission Global Cerebral Edema

Two studies compared cerebral metabolic changes in patients with and without global cerebral edema (GCE) diagnosed by CT-imaging of the brain. Helbok et al. found an association between a higher frequency of metabolic crisis (significantly higher LPR, lower brain glucose levels) and GCE (15). Zetterling et al. described a pattern of cerebral hypermetabolism (higher lactate and pyruvate levels, no significant differences in LPR and glucose levels) in GCE patients (21). Despite this discrepancy, these findings indicate altered brain energy metabolism in SAH patients with GCE. Intracranial hypertension (ICP > 20 mmHg), often a result of brain edema or focal lesions, was associated with a pathologically elevated LPR (>40) and significantly lower CMD-glucose levels (19).

### The Clinical Use of CMD as a Marker of Cerebral Hypoperfuison and DCI in SAH Patients

Delayed cerebral ischemia occurs in up to 30% of SAH patients, mostly between 4 and 10 days after the hemorrhage, and was defined by an international group of experts as either clinical deterioration or cerebral infarction not attributable to other causes (83). In the literature published before 2010, we found a considerable heterogeneity in the definition of delayed ischemia after SAH. Detailed information on the definition used in individual trials is given in Table 4.

Several studies investigated metabolic changes associated with parameters of cerebral perfusion, including CBF, CPP, and imaging surrogates. In summary, a negative correlation between CBF and CMD-lactate, CMD-LPR, CMD-glutamate, and CMD-glycerol has been described, while CMD-pyruvate and CMD-glucose are commonly positively correlated with CBF (25, 31, 32, 35, 38–40). Other studies focused on DCI and found increases in CMD-lactate and CMD-glutamate as early sensitive markers (17). Metabolic derangement with increasing CMD-LPR (> 40) and decreasing CMD-glucose (< 0.7 mmol/l) may occur up to 16 h before DCI onset (26, 32). In the following, we give detailed information on some studies, all studies are listed in Table 4.

Schmidt et al. found an association of CPP < 70 mmHg with a higher incidence of metabolic crisis (CMD-LPR > 40 and CMD-glucose levels < 0.7 mmol/l) and further worsening of brain metabolism at lower CPP values (41). Similarly, a higher CMD-LPR and increased episodes of metabolic distress (CMD-LPR > 40) were observed at a CPP < 60 mmHg in another study including 19 SAH patients (23). However, it is important to elaborate that a high CMD-LPR may also indicate metabolic distress in the absence of ischemia. In this regard, Jacobsen et al. defined an elevated LPR (>30), together with pyruvate levels within normal range (>70 μmol/l) as mitochondrial dysfunction and found this pattern to be 7.5-fold more common than metabolic changes indicative for cerebral ischemia (LPR > 30 and CMD-pyruvate < 70 μmol/l) (28). Mitochondrial dysfunction was moreover associated with higher levels of CMD-glucose and lower levels of CMD-glutamate and CMD-glycerol compared to ischemic episodes.

In poor-grade SAH patients requiring sedation and mechanical ventilation, neurological deterioration may not be detected. In these patients, CMD provides useful information on the metabolic state of the injured brain and may even indicate metabolic changes before DCI occurs. Sarrafzadeh et al. reported higher levels of CMD-lactate and CMD-glutamate in patients with DCI compared to those who did not develop DCI already on day 1 and throughout the first week after the bleeding (17). A higher CMD-LPR was observed from day 3 on (17). This is in line with findings by Nilsson et al., who concluded that CMD-lactate and CMD-glutamate may be the earliest and most sensitive markers of ischemia, followed by the CMD-LPR and CMD-glycerol (30). Furthermore, lower brain glucose levels during the first 3 days after SAH were reported in DCI patients, despite higher blood glucose concentrations when compared to patients who did not develop DCI (29).

Changes in CMD parameters were observed even 12–16 h before the occurrence of DCI and included a significant increase in CMD-LPR to values >40 and decreases in CMD-glucose to levels <0.7 mmol/l (26, 32, 37). At a comparable time point, also elevations of CMD-lactate, CMD-glutamate, and CMD-glycerol were reported (43, 44). The sensitivity of the method is limited due to the local measurement. Therefore, ischemia occurring in brain tissue distant to the monitoring catheter may not be detected (26, 37). Lack of specificity results from metabolic distress secondary to non-ischemic CMD-LPR elevations and hyperglycolytic (non-ischemic) lactate increase (28, 72). Nevertheless, CMD had a higher specificity for predicting DCI than transcranial ultrasound and conventional angiography (44). Unfortunately, most studies do not report on the effect of current treatment strategies for DCI (induced hypertension, intraarterial spasmolysis). Sarrafzadeh et al. reported a decrease in CMD-glutamate levels, but no change in other parameters, associated with “triple-H therapy” (17). Further research focusing on metabolic changes following such interventions is surely warranted.

In summary, studies assessing CBF directly in the region around the CMD probe revealed a highly consistent metabolic pattern of increased CMD-lactate, CMD-glutamate, CMD-glycerol levels, and CMD-LPR during episodes of hypoperfusion, whereas CMD-glucose and CMD-pyruvate levels were positively correlated with CBF. CMD-lactate and CMD-glutamate are early and sensitive markers of impending DCI, but lack of specificity. However, CMD parameters showed a higher specificity for predicting DCI than transcranial ultrasound and conventional angiography (37, 43, 44). Regarding trend analyses, a CMD-LPR increase above 40 and decreasing CMD-glucose concentrations preceded the occurrence of delayed ischemia by several hours (26, 32); however, ischemia that occurred remote from the CMD probe or in the contralateral hemisphere was not detected (26).

### CMD in Monitoring Treatment Effects

Several studies have investigated the effect of pharmacological and non-pharmacological interventions on cerebral metabolism as summarized in Table 5. Studied interventions included the treatment of intracranial hypertension, either with osmotherapy (45, 46), by cerebrospinal fluid drainage *via* external ventriculostomy (53), or by decompressive craniectomy (49, 50), fever treatment with diclofenac or targeted temperature management (51, 52, 54), the management of anemia and administration of packed red blood cells (47, 48, 52), and the administration of erythropoietin or verapamil (55, 56). The impact of enteral nutrition and insulin on brain metabolism will be discussed in the next chapter.

### CMD and Systemic Glucose Management

There is still an ongoing debate on the optimal systemic glucose target in critically ill patients (84, 85). CMD-glucose levels represent the net effect of delivered glucose and glucose consumption. Little is known about the impact of glucose transport and diffusion in acutely brain injured patients.

Severe hyperglycemia (>200 mg/dl = 11.1 mmol/l) is associated with poor outcome in SAH patients (86). Some studies investigated the association between systemic and brain interstitial glucose levels (Table 6). Oddo et al. described the brain metabolic profile during episodes of “low” (<4.4 mmol/), “tight” (4.4–6.7 mmol/l), “intermediate” (6.8–10 mmol/l), and high blood glucose levels in a mixed population of neurocritical care patients, including 10 patients with SAH. Compared to intermediate systemic glucose levels, tight glycemic control was associated with lower CMD-glucose levels and more episodes of CMD-glucose < 0.7 mmol/l, as well as a higher CMD-LPR and more episodes of metabolic crisis (CMD-LPR > 40 and CMD-glucose concentrations < 0.7mmol/l). Metabolic crisis and CMD-glucose < 0.7 mmol/l were associated with higher hospital mortality (61). The significant association of systemic- and CMD-glucose is supported by the results of other groups (59, 66); however, some studies indicate a poor correlation (63, 67), especially in the injured brain. Decreased CMD-glucose is moreover observed when delivery is reduced (i.e., reduction in CBF) or under conditions of increased consumption (i.e., higher body temperature, seizures and the occurrence of cortical spreading depolarizations) (66, 78, 82).

Pathologically low CMD-glucose levels (<0.7 or <0.6 mmol/l) were associated with poor outcome in SAH patients (41, 61, 63). In the recent consensus statement on the use of CMD, clinical experts suggest to intervene when pathologically low CMD-glucose levels are detected (2). This attempt should only be made with respect to probe location (in healthy-appearing brain tissue on head computed tomography), baseline blood glucose levels, and in the absence of brain ischemia. Proposed interventions targeting higher systemic glucose levels include intravenous or enteral glucose administration and the reevaluation of insulin treatment (2).

While no data on intravenous glucose administration are available, three studies sought to investigate the impact of enteral feeding on cerebral metabolism. Schmidt et al. did not observe a direct relation between CMD-glucose levels and the energy content of the administered enteral nutrition (66). Other groups investigating the temporal association of enteral feeding and the metabolic profile revealed time-related increases in CMD-glucose levels not affecting other CMD parameters (58, 59). CMD-glucose levels even increased from critically low (<0.7 mmol/l) levels at baseline independent of probe location (59).

Insulin treatment was associated with a decrease of CMD-glucose independent of serum glucose levels, resulting in a higher incidence of critically low CMD-glucose levels (<0.6 mmol/l) (64, 67), especially under conditions of cerebral metabolic distress (LPR > 40) (66). Moreover, rapid reductions in serum glucose concentrations were associated with a decrease in CMD-glucose (57). In line with this, a higher serum glucose variability was associated with a higher rate of cerebral metabolic distress (60). In summary, tight glycemic control may be associated with pathologically low CMD-glucose levels (neuroglucopenia) in critically ill patients with acute neurologic injury. If brain metabolic monitoring indicates critically low glucose levels (i.e., <0.2 mmol/l) targeting a more liberal glucose regimen (110–150 mg/dl or up to 180 mg/dl) may be indicated.

### CMD and Outcome

Studies reporting an association of CMD parameters with patient outcome are summarized in Table 7. The largest study includes 182 SAH patients and found higher CMD-LPR and CMD-glutamate values during the first week after SAH being significantly and independently associated with poor functional outcome after 12 months (50). Patients with poor functional 3–6 months outcome had significantly more episodes of CMD-LPR > 40 and CMD-glutamate levels > 10 μmol/l compared to those with favorable outcome (71). An elevated CMD-LPR was also associated with hospital mortality (57). Pathologically low CMD-glucose levels < 0.7 mmol/l were observed more often in patients with poor outcome and in those who died (41, 61, 63). Elevated CMD-lactate is a less specific marker for neuroprognostication, as both, associations with good (in a hyperglycolytic context) and poor (due to hypoxia) outcome were reported (72).

Trend analysis is important as a shift to hypermetabolism, indicated by an increase in both, CMD-lactate and CMD-pyruvate levels, was observed in patients with favorable outcome. Persistent low CMD-pyruvate levels without increase to normal values were associated with poor outcome (69). Persistent low CMD-glutamate levels were associated with good functional outcome, whereas increases at day 1 and day 7 were associated with poor outcome (74).

## Discussion

This review demonstrates that CMD is a powerful tool providing almost continuous brain metabolic information at bedside. Based on the published literature, pathological changes in brain metabolism are associated with disease severity, mechanisms of primary and secondary brain injury, and poor long-term outcome after SAH.

As summarized in this review, the major limitation of the published literature is that CMD monitoring was reported mostly in small single-center trials of neurological and neurosurgical ICUs with extensive experience in the use of CMD as adjunct to other multimodal neuromonitoring techniques. In many centers, implementation of CMD as a clinical tool is still limited due to financial constraints and the lack of evidence to improve patient’s outcome. Future trials may address this gap in the literature by targeting brain metabolic parameters as endpoints to improve brain homeostasis as no monitoring tool can improve patient’s outcome unless coupled with a therapeutic intervention. On the other hand, there is a need to re-define commonly used outcome parameters and to move from a simplistic functional outcome score to a more sophisticated approach including neuropsychological testing, quality of life measures, and brain tissue outcome.

The invasiveness of CMD limits its application to poor-grade SAH patients. This implicates that this review merely summarizes brain metabolic changes of unconscious patients and results are not generalizable to all clinical grades. Based on the recently published consensus statement, the use of CMD is recommended in poor-grade SAH patients with prolonged ventilation and patients with secondary neurologic deterioration requiring mechanical ventilation.

Despite these limitations metabolic monitoring in the early and subacute phase after SAH provides insight into pathophysiological mechanisms of primary and secondary brain injury on the brain tissue level. As shown in our review, the detection of impeding ischemia is the most extensively studied application of CMD in SAH patients. CMD has been shown to have the potential to be used as early warning tool of brain tissue ischemia hours before the insult (17, 43, 44), even if clinically silent (26). In this regard, it is important to mention that we recognized a large variability in the definition of DCI throughout the published literature. Although more homogeneity was detected after the year 2010 when Vergouwen et al. defined DCI based on clinical and/or radiographic criteria (83), earlier studies are difficult to compare as information on radiographic evidence of new infarctions related to vasospasm were commonly not reported.

Another limitation is that microdialysis probe location differed in individual studies by means of targeting either brain tissue ipsilateral to the bleeding aneurysm or the contralateral hemisphere and monitoring the cortical gray matter vs. subcortical white matter. Furthermore, probe location was rarely adequately addressed as covariate in multivariate models, which limits the interpretation of results and conclusions. It is important to mention that brain chemistry can only be interpreted correctly based on the relation to focal injury on brain imaging (“normal appearing brain tissue” vs. “perilesional” vs. “intralesional”) (2). As recommended by clinical experts, future trials reporting microdialysis data should at least include information on catheter location, the catheter type used, perfusion fluid composition, the perfusion fluid flow rate, and time from ictus to monitoring (2).

The interpretation of CMD-derived information requires profound knowledge of the complex underlying pathophysiology. As shown in Table 1, metabolic changes may be ambiguous, as similar patterns may indicate different underlying pathophysiological processes. However, taking into account, the occurrence of specific patterns in relation to the bleeding onset may help to discriminate between early and late onset ischemic patterns or patterns of mitochondrial dysfunction. A clinical guidance is given in Table 1 and may be combined with other neuromonitoring parameters such as brain tissue oxygenation, ICP, and CBF.

Clinical implication of microdialysis monitoring besides the detection of secondary ischemic insults include guidance of systemic glucose management, CPP optimization, defining individual transfusion thresholds and monitoring of brain chemistry during pharmacological and non-pharmacological interventions (41, 49, 51, 52, 57, 61, 63, 67).

The only treatment decision based on changes in brain metabolism currently recommended by clinical experts is the treatment of low cerebral glucose taking into account baseline systemic glucose concentration, catheter location, and the etiology of neuroglucopenia. In the knowledge of its potential, there is a need to integrate brain metabolic changes and to define CMD-based endpoints in future clinical trials.

## Conclusion

Cerebral microdialysis is used in the clinical management of severe SAH together with ICP, P_bt_O_2_, and other neuromonitoring parameters. In the knowledge of its limitations, this method provides a novel insight into pathophysiological processes of primary and secondary brain injury. Recent consensus on microdialysis monitoring paves the way for improved protocols and targeted interventions. The major task for future research integrates the prospective evaluation of predefined interventions to improve brain tissue physiology aiming toward a personalized management of SAH patients in the future.

## Author Contributions

RH was involved in the idea, design, data acquisition, article selection, writing, interpretation of data, and final revision of the manuscript. MK was involved in article selection, writing, interpretation of data, and final revision of the manuscript. AS, MG, and VR were involved in the data acquisition, interpretation of data, and final revision of the manuscript. BP, RB, and ES were involved in article selection, writing, interpretation of data, and final revision of the manuscript. All authors read and approved the final version of the manuscript and are accountable for the content, data interpretation, and data accuracy.

## Conflict of Interest Statement

The authors declare that the research was conducted in the absence of any commercial or financial relationships that could be construed as a potential conflict of interest.
